# Non-tunneled versus tunneled dialysis catheters for acute kidney injury requiring renal replacement therapy: a prospective cohort study

**DOI:** 10.1186/s12882-017-0760-x

**Published:** 2017-12-04

**Authors:** Mallika L. Mendu, Megan F. May, Arnaud D. Kaze, Dionne A. Graham, Salena Cui, Margaret E. Chen, Naomi Shin, Ayal A. Aizer, Sushrut S. Waikar

**Affiliations:** 1 Division of Renal Medicine, Brigham and Women’s Hospital, Harvard Medical School, Boston, MA USA; 2 Center for Applied Pediatric Quality Analytics, Boston Children’s Hospital, Boston, MA USA; 3 Internal Medicine Residency Program, Mount Auburn Hospital, Cambridge, MA USA; 4 Department of Radiation Oncology, Brigham and Women’s Hospital, Harvard Medical School, Boston, MA USA; 5 One Brigham Circle, Brigham and Women’s Hospital, Boston, MA 02115 USA

**Keywords:** Acute kidney injury (AKI), Renal replacement therapy (RRT), Non-tunneled dialysis catheter (NTDC), Tunneled dialysis catheter (TDC), Continuous venovenous hemofiltration (CVVH), Intermittent hemodialysis (IHD)

## Abstract

**Background:**

Acute kidney injury requiring renal replacement therapy (AKI-RRT) is associated with high morbidity, mortality and resource utilization. The type of vascular access placed for AKI-RRT is an important decision, for which there is a lack of evidence-based guidelines.

**Methods:**

We conducted a prospective cohort study over a 16-month period with 154 patients initiated on AKI-RRT via either a non-tunneled dialysis catheter (NTDC) or a tunneled dialysis catheter (TDC) at an academic hospital. We compared differences in renal replacement delivery and mechanical and infectious outcomes between NTDCs and TDCs.

**Results:**

Patients who received TDCs had significantly better RRT delivery, both with continuous venovenous hemofiltration (CVVH) and intermittent hemodialysis (IHD), compared to patients who received NTDCs; these findings were confirmed after multivariable adjustment for AKI-specific disease severity score, history of chronic kidney disease, renal consult team, and AKI cause. In CVVH and IHD, the median venous and arterial blood flow pressures were significantly higher with TDCs compared to NTDCs (*p* < 0.001). Additionally for CVVH, the median number of interruptions per catheter was higher with NTDCs compared to TDCs (Rate Ratio (RR) 2.7; *p* < 0.001), and for IHD, a higher median blood flow was seen with TDCs (*p* < 0.001). There were a significantly higher number of mechanical complications with NTDCs (RR 13.6 *p* = 0.001). No significant difference was observed between TDCs and NTDCs for positive blood cultures per catheter.

**Conclusions:**

Compared to NTDCs, TDCs for patients with AKI-RRT had improved RRT delivery and fewer mechanical complications. Initial TDC placement for AKI-RRT should be considered when not clinically contraindicated given the potential for improved RRT delivery and outcomes.

**Electronic supplementary material:**

The online version of this article (10.1186/s12882-017-0760-x) contains supplementary material, which is available to authorized users.

## Background

Acute kidney injury (AKI) affects up to 18% of hospitalized patients [1] and is associated with significant morbidity, mortality, and resource utilization [1–4]. In some individuals, AKI is life threatening and requires the initiation of renal replacement therapy (RRT), most commonly by intermittent hemodialysis (IHD) or continuous renal replacement therapy (CRRT). IHD and CRRT require vascular access to the internal jugular, subclavian or femoral veins to support the high blood flow rates required for treatment. Unlike RRT for end stage renal disease (ESRD), RRT for AKI is most commonly performed using temporary non-tunneled dialysis catheters (NTDC) placed at the bedside. By contrast, tunneled dialysis catheters (TDC), placed in the radiology department with fluoroscopic guidance [5–7], are commonly used in patients with ESRD who do not have functioning arteriovenous fistulas or grafts.

Compared to TDCs, NTDCs have a number of disadvantages. NTDCs have a 5-fold higher rate of infection than TDCs, which have subcutaneous tunnels that increase the distance from the skin to the bloodstream [8, 9]. NTDCs also have a greater likelihood of inadequate blood flow rate, due to the positioning of the catheter tip in the vena cava instead of the right atrium [10, 11]. Nevertheless, NTDCs are the most commonly used catheters for RRT in AKI. In fact, the Kidney Disease: Improving Global Outcomes (KDIGO) guidelines suggest using NTDCs rather than TDCs for vascular access in AKI (level of evidence, 2D) primarily for logistical reasons – namely, ease of insertion and timeliness [4]. The need for urgent bed-side initiation of RRT is one setting in which NTDC placement is more practical, because fluoroscopic guidance is not required as it is for TDC placement. However, in some individuals with AKI that is not immediately life-threatening, TDCs can be placed as the initial choice for vascular access. TDCs are also recommended to replace NTDCs for more prolonged vascular access in severe AKI that requires ongoing RRT for greater than 1 week [12].

At our institution, TDCs are used commonly as the initial choice for vascular access if the need for RRT is expected to be greater than 1 week, and if there are no contraindications to TDC placement. Very little published data are available to compare the performance of TDCs versus NTDCs in AKI requiring RRT (AKI-RRT). The relatively frequent use of TDCs for AKI-RRT at our institution allowed us to compare the outcomes of NTDCs versus TDCs. We conducted a prospective cohort study comparing procedural and clinical outcomes in patients receiving TDCs versus NTDCs. We were specifically interested in comparing rates of bloodstream infections, mechanical complications, and adequacy of RRT delivery in TDCs versus NTDCs when used for RRT in AKI.

## Methods

### Study design and patient population

We prospectively collected data from 154 individuals who were started on RRT for AKI between December 2013 to May 2015 at Brigham and Women’s Hospital (BWH), a tertiary care academic hospital in Boston, MA. The Institutional Review Board approved data collection and analysis of this study. Inclusion criteria included adult patients hospitalized at BWH who required RRT for treatment of AKI. Exclusion criteria included chronic kidney disease (CKD) stage 5 or ESRD, a history of kidney transplantation, pre-existing vascular access (i.e. arteriovenous fistula or arteriovenous graft), outside hospital transfers with an existing catheter for RRT, and placement of dialysis catheters in the operating room after surgery.

### Data sources and collection

We identified all patients with AKI requiring vascular access by reviewing inpatient renal consult patient lists and electronic orders for hemodialysis or CRRT initiation. At our institution CRRT is performed with continuous venovenous hemofiltration (CVVH). We prospectively collected data on demographics, comorbidities, laboratory data, and microbiology results on a daily basis. We abstracted data from procedure notes to identify procedural complications. Similarly, CVVH and IHD data (blood flows, venous and arterial pressures, total time of RRT per session, and number of interruptions during RRT) were gathered prospectively on a daily basis from RRT flowsheets and RRT nursing notes. Patients were followed throughout the entirety of the hospitalization (regardless of whether they moved from the intensive care unit (ICU) to a floor bed or vice versa), until hospital discharge or death.

### Statistical analyses

Baseline patient characteristics were described using standard descriptive statistics and compared at the patient level using analysis of variance, chi-square tests, and Wilcoxon Rank-sum tests as appropriate. Continuous variables related to catheter performance were compared by fitting mixed effect models using an unstructured covariance with two levels of clustering: study subjects and catheters. We adjusted for history of chronic kidney disease, renal consult team (floor vs. intensive care unit), AKI cause (acute tubular necrosis (ATN) versus other), and AKI-specific disease severity at the time of enrollment. Disease severity was estimated using the AKI–specific disease severity equation by Demirjian et al. derived in a randomized controlled trial (RCT) of patients with AKI requiring RRT [13]. This AKI severity score has been shown to have improved performance for mortality prediction in patients with AKI compared with Sequential Organ Failure Assessment and Acute Physiology and Chronic Health Evaluation II scores [13]. Laboratory data for the disease severity score were missing on enrollment in 16 of 154 patients (most commonly arterial PO2). Missing data for AKI disease severity were imputed as normal values, given the likelihood that missing data on variables, such as pH, indicated low clinical suspicion for abnormal values. Count data related to catheter complications were analyzed with generalized linear mixed models assuming a Poisson distribution, with adjustment for AKI disease severity, history of chronic kidney disease, renal team and AKI cause (ATN versus other) at the time of study enrollment (Table 3). All statistical analyses were performed with SAS 9.4 (SAS Institute, Cary, NC).

## Results

### Patient demographics and clinical characteristics

We enrolled 154 patients with AKI requiring vascular access for RRT. ATN was the most common cause of AKI (55.8%). Seventy seven patients were treated with NTDCs only, 35 with TDCs only, and 42 with both NTDCs and TDCs. In total, 140 NTDCs and 80 TDCs were placed. Additional file 1: Table S1 shows details of catheter length, anatomical site, and catheter tip positioning. There were significant differences in the types of renal replacement therapy received by the three groups (Table 1). Patients who received only NTDCs were more often treated with CVVH and in the ICU than those who received TDCs or both types of catheters. Patients who received only TDCs were more often treated with IHD and outside of the ICU than those who received NTDCs or both. In-hospital mortality was significantly higher in those who received only NTDCs (74.0%) than TDCs (22.9%) or both (14.3%).

**Table 1:** Patient characteristics according to type of catheter used for AKI requiring renal replacement therapy

	NTDC only	TDC only	Both NTDC and TDC	*p*-value
Number of patients	77	35	42	–
Number of catheters	91	36	49 non-tunneled, 44 tunneled	–
Age, mean ± SD	61.1 ± 15.1	62.4 ± 15.6	62.6 ± 13.2	0.84
Female, %	40.3	48.6	42.9	0.71
Race, %				0.27
White	81.8	77.2	90.5	
Black	5.2	14.3	4.8	
Hispanic	2.6	5.7	4.8	
Other	10.4	2.9	0	
AKI severity of disease^a^ score, mean ± SD	26.4 ± 7.4	15.8 ± 5.1	21.5 ± 6.6	<0.001
Charlson score, median (IQR)	2 (1–3)	3 (2–5)	3 (1–5)	0.52
Hypertension, %	62.3	71.4	71.4	0.49
Diabetes mellitus, %	37.7	40.0	40.5	0.95
Chronic kidney disease, %	16.9	34.3	38.1	0.02
Congestive heart failure, %	19.5	14.3	35.7	0.05
Coronary artery disease, %	31.2	40.0	31.0	0.62
Hyperlipidemia, %	40.3	34.3	45.2	0.62
Malignancy, %	32.5	31.4	23.8	0.60
Cause of AKI				<0.001
ATN	63.6	34.3	59.5	
Other^b^	36.4	65.7	40.5	
Primary team, %				<0.001
MICU	48.1	8.6	40.5	
CCU	22.1	5.7	11.9	
Medicine	3.9	40	9.5	
Oncology	5.2	17.1	11.9	
Other	20.8	28.6	26.2	
Renal team, %				<0.001
Consult	7.8	77.1	31.0	
ICU	90.1	22.9	69.1	
Type of RRT, %^c^				<0.001
IHD only	16.9	91.4	42.9	
CVVH only	58.4	5.7	4.8	
Both IHD and CVVH	24.7	2.9	52.4	
Number of days on RRT, Median (IQR)^d^	6 (3–11)	8 (4–13)	17 (10–28)	<0.001
In-hospital mortality, %	74.0	22.9	14.3	<0.001

*AKI* Acute kidney injury; *ATN* Acute tubular necrosis; *CVVH* Continuous veno-venous hemofiltration; *CCU* Coronary care unit; *ICU* Intensive care unit; *IHD* Intermittent hemodialysis; *IQR* Interquartile range; *MICU*, Medical intensive care unit; *NTDC* Non-tunneled dialysis catheter; *RRT* Renal replacement therapy; *SD* Standard deviation; *TDC* tunneled dialysis catheter

^a^Risk equation by Demirjian et al. [13] calculated at time of initiation of RRT

^b^Other common causes of AKI in descending order include: other (35.7%) pre-renal azotemia (3.3%), tumor lysis (3.3%), and acute interstitial nephritis (1.3%)

^c^Type of RRT, % - type of modality of RRT patient received while in the hospital

^d^Number of days on RRT – in-hospital number of days on RRT

### Renal replacement delivery parameters

We compared metrics of RRT delivery and performance between NTDCs and TDCs in multivariable-adjusted analyses at the catheter level (Table 2). Compared to TDCs, NTDCs had statistically significantly lower median venous and arterial access pressures. TDCs allowed for significantly higher mean blood flow rates than NTDCs when used for IHD but not for CVVH (blood flows for CVVH are prescribed at 250 ml/min as standard at our institution). The rate of interrupted delivery of CVVH due to catheter malfunction was more than twice as high for NTDCs than TDCs (adjusted rate ratio of 2.7; *p* < 0.001); other metrics of adequacy of CVVH including hours of treatment and mean BUN levels were not different across the two types of catheters. Results were qualitatively unchanged in analyses restricted to nontunneled and tunneled catheters placed in the internal jugular vein (data not shown).

**Table 2:** Renal replacement therapy delivery parameters in non-tunneled versus tunneled dialysis catheters used for AKI requiring renal replacement therapy

	Adjusted analyses^b^		
RRT Delivery Parameter	NTDC (*n* = 140)^a^	TDC (*n* = 80)^a^	*p*-value
CVVH			
Blood flow ml/min, mean ± SE	242.8 ± 4.9	246.3 ± 6.2	0.44
Median venous access pressure, mean ± SE	87.9 ± 7.4	121.1 ± 9.7	<0.001
Median arterial access pressure, mean ± SE	58.3 ± 6.4	80.3 ± 8.2	<0.001
Hours of CVVH, mean ± SE	17.8 ± 1.2	17.9 ± 1.5	0.91
BUN, mean ± SE	59.8 ± 9.5	58.6 ± 11.0	0.86
Rate Ratio of interruptions per catheter^c^	2.7 (1.7–4.3)	1 (ref)	<0.001
IHD			
Blood flow ml/min, mean ± SE	319.0 ± 12.8	368.2 ± 13.0	<0.001
Median venous pressure, mean ± SE	84.6 ± 8.9	145.1 ± 9.1	<0.001
Median arterial pressure, mean ± SE	108.3 ± 10.5	179.3 ± 10.7	<0.001

*BUN* Blood urea nitrogen; *CVVH* Continuous veno-venous hemofiltration; *IHD* Intermittent hemodialysis; *NTDC* Non-tunneled dialysis catheter; *RRT* Renal replacement therapy; *SE* Standard error; *TDC* Tunneled dialysis catheter

^a^Number of catheters

^b^Adjusted for ATN risk score, history of chronic kidney disease, renal team and AKI cause (ATN vs others)

^c^From mixed effect models

### Complications

**Fig. 1:**
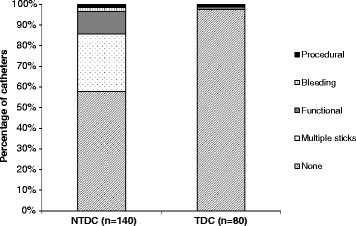
Mechanical complications in non-tunneled versus tunneled dialysis catheters used for AKI requiring renal replacement therapy. Abbreviations: NTDC, non-tunneled dialysis catheter; TDC, tunneled dialysis catheter. Procedural complications included need to pull back catheter, cuff migration, and missing the internal jugular vein. Bleeding complications included bleeding and hematoma formation. Functional complications included catheter malfunction, clotting, and need for exchange

Overall, in the entire study cohort, there were 311 blood cultures drawn of which 26 were positive, and a total of 68 mechanical complications (45 catheter placements that required more than a single stick, 13 stopped working, 3 clotting incidents, 2 bleeding incidents, 2 placement complications requiring re-positioning, 1 hematoma, 1 need for exchange, and 1 missed internal jugular vein). Only 2 of 80 TDCs had mechanical complications, compared to 92 of 140 NTDCs (Fig. 1). We compared the relative rates of both infectious and mechanical complications in NTDCs versus TDCs in multivariable-adjusted generalized linear mixed effects regression models at the catheter level (Table 3). Compared to TDCs, NTDCs had significantly higher adjusted rates of cultures drawn per catheter but not different rates of positive blood cultures. NTDCs had significantly higher rates of mechanical complications and overall complications (mechanical and positive cultures) than TDCs (RR 13.6, 95% CI 2.9–63.0, *p* = 0.001). The difference in mechanical complication rate was even more pronounced when incorporating multiple sticks, (RR 69.1, 95% CI 16.6–288.2, *p* < 0.001). The number of catheters placed per patient was higher in patients who initially had an NTDC compared to those who initially had a TDC. Results were qualitatively unchanged in analyses restricted to nontunneled and tunneled catheters placed in the internal jugular vein (data not shown).

**Table 3:** Complications in non-tunneled versus tunneled dialysis catheters used for AKI requiring renal replacement therapy

Complication outcome	Adjusted rate ratio,^a^ NTDC vs TDC (95% CI)	*p*-value
Blood cultures		
Cultures drawn per catheter	2.1 (1.7–2.8)	<0.001
Positive cultures drawn per catheter	1.4 (0.6–3.4)	0.41
Mechanical complications		
Mechanical complications (excluding multiple sticks)	13.6 (2.9–63.0)	0.001
Mechanical complications (including multiple sticks)	69.1 (16.6–288.2)	<0.001
All complications		
Positive cultures and mechanical complications (excluding multiple sticks)	3.3 (1.6–6.8)	<0.001
Positive cultures and mechanical complications (including multiple sticks)	12.5 (6.5–24.0)	<0.001
Number of catheters per patient	1.8 (1.2–2.6)	0.002

*CI* Confidence interval; *NTDC* Non-tunneled dialysis catheter; *TDC* Tunneled dialysis catheter

^a^Adjusted rate ratios for complications with non-tunneled versus tunneled catheters (reference group), from mixed effects models adjusted for ATN risk score, history of chronic kidney disease, renal team, and AKI cause (ATN vs others)

## Discussion

The primary findings in this prospective study comparing types of vascular access for AKI-RRT are that TDCs have substantially fewer complications and better performance characteristics than NTDCs, even after multivariable adjustment for disease severity. Compared to NTDCs, TDCs had fewer mechanical complications, fewer blood draws for suspected infections, and superior performance characteristics such as blood flow rates during IHD. These clinical outcomes may have a meaningful impact on patients, as reduced dialysis delivery could impact survival, and mechanical complications can range from discomfort to life-threatening sequelae. Our study is novel in its prospective tracking of catheter placement for RRT in AKI patients and examination of clinically relevant outcomes.

Few studies have compared outcomes of NTDCs versus TDCs in AKI patients requiring RRT. Klouche et al. performed a small randomized controlled trial of NTDCs versus femoral TDCs in 30 patients with AKI, and found lower rates of catheter-related bacteremia as well as significant improvements in dialysis adequacy with TDCs compared to NTDCs [11]. However, it is difficult to extrapolate these results as tunneled femoral catheters are rarely used in clinical practice in the United States. Weijmer et al. performed a retrospective study involving both AKI and ESRD patients that showed significantly higher rates of infection in patients with NTDC (15.6–20.2/ 1000 catheter days) compared to TDC (2.9/ 1000 catheter days). However, this study included ESRD patients. The reasons for the superior outcomes of tunneled catheters relate to inherent characteristics of the catheters, namely length (longer catheters to allow for tunneling) and positioning (within the right atrium). In our study, the significant majority of tunneled catheters were 23 cm or longer and were positioned in the right atrium (see Additional file 1: Table S1). We also found consistent results in analyses restricted to nontunneled and tunneled catheters placed in the internal jugular vein, suggesting that inherent characteristics of NTDCs versus TDCs drive our findings.

Our study raises a number of important issues related to catheter placement for acute RRT. The rationale for consensus guidelines recommending initial NTDC placement for acute RRT is presumably due to the ease of arranging placement (i.e., can be performed at the bedside, with little preparation or scheduling) and ease of removal (i.e., without dissection of the cuff). However, our findings suggest that it may be worth pursuing initial TDC if feasible, due to improvements in performance and safety of TDCs. For patients facing critical illness, minimizing the risk of a hematoma or pneumothorax and improved delivery of dialysis may be clinically significant. In addition, almost a third of patients obtained both a NTDC and TDC during their hospitalization, with a mean duration of RRT of 17 days (interquartile range of 10–28). Therefore, a significant percentage of patients have to undergo at least two catheter placement procedures during hospitalization, when likely one procedure would have been sufficient. In one study involving TDCs for AKI-RRT, 80% of patients required RRT for greater than 1 week [14]. Similarly, in the Acute Renal Failure Trial Network study, 73% of patients had no recovery of kidney function by day 28 of hospitalization [15]. It is also notable in the current study that TDCs alone were utilized in 23% of patients with AKI-RRT in the ICU, highlighting that placement at the bedside is not the only option for many patients who are critically ill.

Of course there are a number of clinical scenarios which preclude TDC placement: 1) when urgent catheter placement is warranted due to life-threatening hyperkalemia or toxic ingestion; 2) clinical instability requiring bedside-only placement; and 3) lack of accessible internal jugular vasculature for TDC placement. In addition, implementing a TDC-first policy could require the creation of or expansion of interventional radiology or interventional nephrology access to ensure optimal delivery of care. Published guidelines emphasize that TDCs are more technically challenging to place: “...a cumbersome procedure that requires expertise... and effort...The removal is also technically more difficult [4].”

Our study has a number of limitations that should be considered in evaluating its findings. First, we used indirect measures of RRT delivery including blood flows, arterial and venous pressures, hours of treatment, median BUN, and number of interruptions in treatments. It is notable that for CVVH there was no difference in the number of hours of CVVH administered for TDC versus NTDCs or daily median BUN. Second, NTDCs were generally placed by nephrology fellows and attendings, whereas TDCs were placed by interventional radiologists, raising the possibility that operator characteristics could have been responsible for some of the observed differences in complications rates. Third, patients with NTDCs had higher mortality than patients with TDCs, so some of the observed differences in outcomes may have been confounded by differences in disease severity. Finally, the study was conducted at a single academic center with a relatively limited sample size, which limits its generalizability. Randomized controlled trials are needed to definitively test whether a TDC-first policy may improve outcomes in patients with AKI-RRT.

## Conclusions

In conclusion, we have shown that TDC placement for AKI requiring RRT may lead to fewer mechanical complications and improvements in metrics of RRT delivery. TDCs should be considered as the initial catheter of choice in AKI-RRT in the appropriate clinical setting when not contraindicated.

## Additional file

Additional file 1: Table S1. Characteristics of non-tunneled versus tunneled dialysis catheters used for AKI requiring renal replacement therapy. (DOCX 16 kb)

## Abbreviations

AKI: Acute kidney injury
AKI-RRT: Acute kidney injury requiring renal replacement therapy
ATN: Acute tubular necrosis
BUN: Blood urea nitrogen
BWH: Brigham and Women’s Hospital
CCU: Coronary care unit
CI: Confidence interval
CKD: Chronic kidney disease
CRRT: Continuous renal replacement therapy
CVVH: Continuous venovenous hemofiltration
ESRD: End stage renal disease
ICU: Intensive care unit
IHD: Intermittent hemodialysis
IQR: Interquartile range
KDIGO: Kidney Disease: Improving Global Outcomes
MICU: Medical intensive care unit
NTDC: Non-tunneled dialysis catheter
RCT: Randomized controlled trial
RR: Rate Ratio
RRT: Renal replacement therapy
SD: Standard deviation
SE: Standard error
SVC: Superior Vena Cava
TDC: Tunneled dialysis catheter

## References

[1] Chertow GM, Burdick E, Honour M, Bonventre JV, Bates DW (2005). Acute kidney injury, mortality, length of stay, and costs in hospitalized patients. J Am Soc Nephrol.

[2] Zeng X, McMahon GM, Brunelli SM, Bates DW, Waikar SS (2014). Incidence, outcomes, and comparisons across definitions of AKI in hospitalized individuals. Clin J Am Soc Nephrol.

[3] Allegretti AS, Steele DJR, David-Kasdan JA, Bajwa E, Niles JL, Bhan I (2013). Continuous renal replacement therapy outcomes in acute kidney injury and end-stage renal disease: a cohort study. Crit Care.

[4] Kidney disease: improving global outcomes (KDIGO) acute kidney injury work group. KDIGO clinical practice guideline for acute kidney injury. Kidney inter., Suppl. 2012;2:1–138. http://www.kdigo.org/clinical_practice_guidelines/pdf/KDIGO%20AKI%20Guideline.pdf.

[5] Weijmer MC, Vervloet MG, ter Wee PM (2004). Compared to tunnelled cuffed haemodialysis catheters, temporary untunnelled catheters are associated with more complications already within 2 weeks of use. Nephrol Dial Transplant.

[6] Van Der Meersch H, De Bacquer DD, Vandecasteele SJ, Van den Bergh B, Vermeiren P, De Letter J, De Vriese AS (2014). Hemodialysis catheter design and catheter performance: a randomized controlled trial. Am J Kidney Dis.

[7] Canaud B, Desmeules S, Klouche K, Leray-Moragues H, Beraud JJ (2004). Vascular access for dialysis in the intensive care unit. Best Pract Res Clin Anaesthesiol.

[8] Vats HS (2012). Complications of catheters: tunneled and nontunneled. Adv Chronic Kidney Dis.

[9] Raad I (1998). Intravascular catheter-related infections. Lancet.

[10] Asif A, Cherla G, Merrill D, Cipleu CD, Briones P, Pennell P (2005). Conversion of tunneled hemodialysis catheter-consigned patients to arteriovenous fistula. Kidney Int.

[11] Klouche K, Amigues L, Deleuze S, Beraud JJ, Canaud B (2007). Complications, effects on dialysis dose, and survival of tunneled femoral dialysis catheters in acute renal failure. Am J Kidney Dis.

[12] National Kidney Foundation K/DOQIclinical practice guidelines for vascular access: update 2000. Am J Kidney Dis. 2001;37:S137–81. 10.1016/s0272-6386(01)70007-8 11229969

[13] Demirjian S, Chertow GM, Zhang JH, O’Connor TZ, Vitale J, Paganini EP, Palevsky PM (2011). VA/NIH acute renal failure trial network: model to predict mortality in critically ill adults with acute kidney injury. Clin J Am Soc Nephrol.

[14] Coryell L, Lott JP, Stavropoulos SW, Mondschien JI, Patel AA, Kwak A, Soulen MC, Solomon JA, Shlansky-Goldberg RD, Nemeth AA, Kobrin S, Rudnick M, Trerotola SO (2009). The case for primary placement of tunneled Hemodialysis catheters in acute kidney injury. J Vasc Interv Radiol.

[15] Palevsky PM, Zhang JH, O'Connor TZ, Chertow GM, Crowley ST, Choudhury D, Finkel K, Kellum JA, Paganini E, Schein RM, Smith MW, Swanson KM, Thompson BT, Vijayan A, Watnick S, Star RA, Peduzzi P, VA/NIH Acute Renal Failure Trial Network (2008). Intensity of renal support in critically ill patients with acute kidney injury. N Engl J Med.

