# Supplementary material for: Non-tunneled versus tunneled dialysis catheters for acute kidney injury requiring renal replacement therapy: a prospective cohort study
Source: BMC Nephrol. 2017 Dec 4;18:351. doi: 10.1186/s12882-017-0760-x (PMC5715550; doi:10.1186/s12882-017-0760-x)
Supplement: Additional file 1: Table S1. — Characteristics of non-tunneled versus tunneled dialysis catheters used for AKI requiring renal replacement therapy. (DOCX 16 kb) [file 12882_2017_760_MOESM1_ESM.docx]

|  | **NTDC** | **TDC** |
| --- | --- | --- |
| **Length (cm)** | | |
| 13.5 | 16 (11.4) | - |
| 15 | 1 (0.7) | - |
| 16 | 41 (29.3) | - |
| 19 | - | 8 (10.0) |
| 20 | 56 (40.0) | - |
| 23 | - | 42 (52.5 |
| 24 | 26 (18.6) | 1 (1.3) |
| 27 | - | 24 (30.0) |
| 31 | - | 5 (6.3) |
| **Location** | | |
| Left femoral | 14 (10.0) | - |
| Left internal jugular | 38 (27.1) | 21 (26.3) |
| Left subclavian | 1 (0.7) | - |
| Right femoral | 21 (15.0) | - |
| Right internal jugular | 66 (47.1) | 59 (73.8) |
| **Position of catheter tip** | | |
| SVC | 87 (82.9) | 1 (1.3) |
| Right atrium^a^ | 14 (13.3) | 79 (98.8) |
| Other^b^ | 4 (3.8) |  |

**Supplementary Table 1: Characteristics of non-tunneled versus tunneled dialysis catheters used for AKI requiring renal replacement therapy.**

Abbreviations: NTDC, non-tunneled dialysis catheter; SVC, superior vena cava; TDC, tunneled dialysis catheter.

a: Includes right atrium and cavoatrial junction

b: Includes no available imaging (3) and brachiocephalic vein (1)
